# Diagnostic accuracy of maternal serum multiple marker screening for early detection of gestational diabetes mellitus in the absence of a gold standard test

**DOI:** 10.1186/s12884-020-03068-7

**Published:** 2020-06-26

**Authors:** Maedeh Amini, Anoshirvan Kazemnejad, Farid Zayeri, Ali Montazeri, Aliakbar Rasekhi, Azam Amirian, Nourossadat Kariman

**Affiliations:** 1 Department of Biostatistics, Faculty of Medical Sciences, Tarbiat Modares University, Tehran, Iran; 2 Proteomics Research Centre and Department of Biostatistics, School of Allied Medical Sciences, Shahid Beheshti University of Medical Sciences, Tehran, Iran; 3 Health Metrics Research Centre, Iranian Institute for Health Sciences Research, ACECR, Tehran, Iran; 4 Department of Midwifery, School of Nursing and Midwifery, Jiroft University of Medical Sciences, Jiroft, Iran; 5 Department of Midwifery and Reproductive Health, School of Nursing and Midwifery, Shahid Beheshti University of Medical Sciences, Tehran, Iran

**Keywords:** Gestational diabetes mellitus, Beta-human chorionic gonadotropin, Unconjugated estriol, Alfa-fetoprotein, Body mass index, Sensitivity, Specificity, Receiver operator characteristic (ROC) curve, Bayesian analysis

## Abstract

**Background:**

Gestational diabetes mellitus (GDM) is associated with adverse diabetic complications for both mother and child during pregnancy. The common Gold Standard (GS) for diagnosis of GDM is 75 g oral glucose tolerance test (OGTT) during 24–28 gestational weeks which seems a little late for any proper intervention. This study aimed to employ the Bayesian latent class models (LCMs) for estimating the early diagnostic power of combination of serum multiple marker in detecting GDM during 14–17 weeks of gestation.

**Methods:**

Data from a sample of 523 pregnant women who participated in gestational diabetes screening tests at health centers affiliated to Shahid Beheshti University of Medical Sciences in Tehran, Iran from 2017 to 2018 were used. The beta-human chorionic gonadotropin (β-hCG), unconjugated estriol (uE3), and alfa-fetoprotein (AFP) values were extracted from case records for all participants. The Bayesian LCMs were applied for estimating sensitivity, specificity, and area under receiver operating characteristic curve (AUC) of combining the three biomarkers’ results in the absence of GS, adjusting for maternal age and body mass index.

**Results:**

The mean (standard deviation) maternal age of the participants was 28.76 (±5.33) years. Additionally, the mean (standard deviation) BMI was 24.57 (±3.22) kg/m^2^. According to the Bayesian model, the cSensitivity, cSpecificity, and cAUC for the optimal composite diagnostic test were estimated as 94% (95% credible interval (CrI) [0.91–0.99]), 86% (95% CrI [0.80–0.92]), and 0.92 (95% CrI [0.87–0.98]), respectively.

**Conclusions:**

Overall, the findings revealed that the combination of uE3, AFP, and β-hCG results might be considered as an acceptable predictor for detecting GDM with a rather high level of accuracy in the early second trimester of pregnancy without a GS.

## Background

The most common medical complication of pregnancy is gestational diabetes mellitus (GDM) which has been defined as any degree of glucose intolerance onset or first recognition during pregnancy [1]. Globally it affects 9.8 to 25.5% of pregnancies worldwide [2]. As such, little is known about the burden of GDM in various parts of the world. Specifically, it is important to note that despite high prevalence of the disease and its mortality in low- and middle-countries rates, there are only a few studies about the burden of GDM in these countries [3, 4]. It is well documented that GDM, as a metabolic disorder, is associated with adverse maternal and neonatal outcomes. For instance, it could increase the incidence of pre-eclampsia, macrosomia, obesity, type 2 diabetes and metabolic syndrome [5, 6]. Therefore, early detection of the disease for preventing the adverse effects is very essential.

The currently available gold standard (GS) for diagnosis of GDM is 75 g oral glucose tolerance test (OGTT) at 24 to 28 gestational weeks. This test has some limitations such as laboratory cost, time consuming nature, drinking a glucose solution and waiting for 2 or 3 h before having the final blood test, taking a series of blood sugar tests over 1 to 3 h, labour-intensive, patient’s need for fasting prior to the test, conflicting results in people from different races and ethnicities, some patient’s intolerance to high amounts of powdered sugar and low reproducibility which can add to the uncertainty in confirming a diabetes diagnosis. Likewise, the OGTT is unable to detect mild glucose intolerance and this deficiency could lead to perinatal adverse effects. Additionally, the 75 g OGTT is not used universally and none of the guidelines provide robust evidence for the reason behind performing OGTT at 24–28 gestational weeks. Nevertheless, one of the most important limitations of the OGTT is the fact that the test is performing in the late second trimester of pregnancy [7–11]. Delayed diagnosis of GDM appears to be the main problem in the prevention of short-term and long-term health consequences for the offspring and increased long-term risk of cardio-metabolic disease in the mothers [12, 13]. However, a number of studies have found that changes in the maternal serum markers that are routinely screened during pregnancy for early detection of adverse pregnancy outcomes and high-risk pregnancies in the current obstetric practice might be helpful in diagnosis of GDM [14]. At present, β-human chorionic gonadotropin (β-hCG), unconjugated Estriol (uE3), and alfa-Fetoprotein (AFP) are known as the triple-marker test that shown to be effective and non-invasive tool for the identification of pregnant women at risk. This test has been validated and become the preferred screening test for Down syndrome and open neural tube defect in the late first trimester or early second trimester of pregnancy [14–18]. Previous studies have indicated the association of maternal serum levels of β-hCG and uE3 or AFP with a variety of problems of pregnancy such as stillbirth, oligohydramnios, polyhydramnios or antepartum haemorrhage, preterm labor-birth and GDM [19]. It is worth to note that according to the literature, the increased levels of β-hCG and AFP or low levels of uE3 are thought to reflect early placental pathology that may be associated with complications later in pregnancy [14].

In clinical practice, for assessing the performance of a new diagnostic test, the result should be compared with the outcome of a gold standard. Ideally, when a gold standard is available, estimating the accuracy measures such as sensitivity, specificity, and area under the receiver operating characteristic (ROC) curve (AUC) is straightforward and without error [20]. However, problems arise when the true disease status of a person could not be identified with certainty due to several reasons including ethical issues, GS test is too expensive, invasive, or it cannot practically be performed, etc. [21]. Some investigators argued that the absence of a GS might lead to misclassification of disease status and biased estimates of tests accuracy parameters. In such situations, obtaining a definitive verification of diagnosis for each subject becomes challenging [22, 23]. Hence, it is vital to use the more advanced statistical techniques for evaluating the performance of the new test when a GS is not available.

In the case of more than a single diagnostic test, it is usually desirable to combine the results of multiple tests into a composite diagnostic test in order to obtain more accurate disease classifications [24, 25]. It is worth to note that combining test results may help clinicians make better diagnostic judgment and increased clinical benefits. In such settings, to evaluate the diagnostic performance, one can compare the diagnostic accuracy of the combined tests as opposed to the accuracy of a single test [26]. Latent class or finite mixture models have been increasingly used to combine the results from multiple diagnostic tests through a statistical model to get estimates of disease prevalence and test accuracy in the absence of a gold standard. Clearly, in this modeling approach, the unobserved disease status serves as a latent variable and observed associations among the diagnostic tests are explained by the latent variable [27]. To estimate accuracy parameters of tests, most of the literature had accomplished within the Bayesian framework. This is a well-established method for robust assessment of diagnostic tests [28–31].

The goal of the current study was to explore whether combining the results obtained from different biomarkers could aid in prediction of GDM between 14 and 17 weeks of gestation when the true disease status is unknown. For this purpose, we applied the Bayesian latent class models (LCMs) for estimating sensitivity, specificity, and AUC adjusting for some confounding factors.

## Methods

### Study population and screening tests

In this research, we utilized the data from 523 pregnant women (aged from 20 to 40 years) who referred to the health centers affiliated to Shahid Beheshti University of medical sciences in Tehran, Iran, for screening GDM from January 2017 to December 2018. A diagnostic two-hour 75 g oral glucose tolerance test was carried out for all pregnant women between 24th and 28th weeks of gestation. Inclusion criteria were women with a singleton pregnancy aged 20–40 years with a gestational age of 24–34 weeks. The exclusion criteria were: having type II diabetes in first-degree relatives, having habitual abortion, having fetal anomalies and macrosomia, intake of medications affecting glucose metabolism, smoking, and drug use. For all women, maternal serum β-hCG, uE3, and AFP levels were measured by a solid-phase, competitive chemiluminescent enzyme immunoassay method as multiples of median (MOM) during 14–17 weeks of gestation. Body weight and height were measured at the same time, in light indoor clothing and without shoes. Body mass index (BMI) was calculated as weight (kg) divided by height squared (m^2^). All pregnant women provided written informed consent. The Ethics Committee of Tarbiat Modares University approved the study.

### Outcome

The main outcome of interest was gestational diabetes mellitus. Here, we emphasize that in the dataset, there was no information about having or not having GDM for each pregnant woman at 14 to 17 gestational weeks. Clearly, the true disease status was not identified in the early second trimester of pregnancy. Hence, it is considered as a latent variable in the applied statistical model.

### Statistical methods

The demographic characteristics of the women were presented using the descriptive statistics such as mean ± standard deviation (SD). The Kolmogorov-Smirnov test was utilized to assess the normality of the distribution for each biomarker’s result.

When the true disease status is unknown, the traditional statistical methods for assessing the diagnostic accuracy of test are not valid. These methods assume the existence of a GS test that has perfect sensitivity and specificity. In the past decades, several studies have been proposed different statistical techniques as a general solution to the problem of not having a GS assessment. Of these, latent class modelling has been extensively used in medical science, specifically in test accuracy research. This modelling approach which relates the observed results of diagnostic test to the latent disease status, can provide valid estimates of accuracy measures in the absence of a perfectly accurate disease status classification [22].

In the current study, the Bayesian latent class model was applied to correctly classify women into clinically meaningful subgroups. Firstly, the fitted model for each of the biomarkers is described in the following paragraph according to a GS (i.e., OGTT) that assumed is not available [28]:

Assume *Y*_*i*_ denotes the results of experimental continuous biomarker for subject *i* (*i* = 1, 2, …, 523). Let *d*_*i*_ be the latent variable that indicates the results of the unobserved gold standard reference test based on disease status of the *i*th individual (1: presence, 0: absence). If biomarker’ scores are normally distributed (even after a suitable transformation), then latent class model can be defines as (without covariate):

1 $$ {\displaystyle \begin{array}{c}{d}_i\sim \mathrm{Bernoulli}\left({\pi}_i\right)\\ {}\left(\left.{Y}_i\right|{d}_i\right)\sim {g}_1{\left(\left..\right|{\mu}_D,{\sigma}_D^2\right)}^{d_i}{g}_2{\left(\left..\right|{\mu}_{\overline{D}},\sigma \frac{2}{D}\right)}^{1-{d}_i}\end{array}} $$

where *μ*_*D*_ and ${\mu}_{\overline{D}}$ are the means, and ${\sigma}_D^2$ and ${\sigma}_{\overline{D}}^2$ are the variances for the normal models of biomarker’ outcome for disease (D) and non-diseased ($\overline{\mathrm{D}}$) populations, respectively. Also, *g*_1_(.) and *g*_2_(.) are the probability density functions $N\left({\mu}_D,{\sigma}_D^2\right)$ and $N\left({\mu}_{\overline{\mathrm{D}}},{\sigma}_{\overline{\mathrm{D}}}^2\right)$, respectively. One the other hand, *π*_*i*_ denotes the probability of a disease such that *P*(*d*_*i*_ = 1) = 1 – *P*(*d*_*i*_ = 0) = *π*_*i*_. Meanwhile, in the absence of a GS, may be the model lacks identifiability. Hence, to achieve model identifiability, we assume that ${\mu}_D>{\mu}_{\overline{\mathrm{D}}}$. Furthermore, to determine how close the distribution of *Y*_D_ to the distribution of ${Y}_{\overline{D}}$, we used the measure (Δ) proposed by choi et al. Note that when Δ is large (near to 0.5 or greater than 0.5), overlapping is increased between diseased and non-diseased group. Under this condition, the proposed method may not work well and convergence problems occur [28, 31]. After obtaining the model parameter estimates, the ROC curve for cutoff values *c* ∈ (−∞, ∞) based on single biomarker can be constructed by plotting $\left(1-\mathrm{spesificity}(c),\mathrm{sensitivity}(c)\right)=\left(1-\Phi \left(\frac{c-{\mu}_{\overline{D}}}{\sqrt{\sigma_{\overline{D}}^2}}\right),1-\Phi \left(\frac{c-{\mu}_D}{\sqrt{\sigma_D^2}}\right)\right)$, where 1- specificity and sensitivity are referred to false positive probability and true positive probability, respectively. In addition, ϕ is the cumulative distribution function of a standard normal for the biomarker’ scores. Finally, the corresponding AUC which is a measure of the overall performance of a diagnostic test, can be calculated as $AUC=\Phi \left(-\frac{\mu_{\overline{D}}-{\mu}_D}{\sqrt{\sigma_{\overline{D}}^2+{\sigma}_D^2}}\right)$. This measure can take on any value between 0 and 1. Notably, the closer AUC is to 1, the better the ability to discriminate between subjects with and without a disease.

In order to estimate *θ* = (*π*, *μ*_*D*_, ${\mu}_{\overline{D}},{\sigma}_D^2,{\sigma}_{\overline{D}}^2\Big),$ we employed Bayesian approach. We assumed non-informative prior distributions for all of the parameters. For *μ*_*D*_ and ${\mu}_{\overline{D}}$, and for $1/{\sigma}_D^2$ and $1/{\sigma}_{\overline{D}}^2$, normal and gamma priors were selected, respectively. Besides, for *π*, dirichlet prior was chosen. Additionally, the Markov chain Monte Carlo (MCMC) method was utilized to obtain the Bayesian estimated parameters according to the posterior distribution. The mean, standard deviation, and 95% credible interval (CrI) as the posterior summary measures were employed. Meanwhile, we applied Monte Carlo (MC) error which is the computational accuracy of the mean. The convergence of the MCMC technique can be assessed by various criterion as well as autocorrelation diagnostic plots. If the autocorrelation within chains is not high, this may be satisfactory evidence for convergence.

Second, we combined the three markers into a single composite diagnostic test based on the model proposed by Yu et al. in 2011 [30]. First, we considered different double linear combination of biomarkers for diagnosis. Then, the linear combination of the three biomarker results was examined. At this stage, for evaluation of classification accuracy of marker combinations, we used covariate-adjusted ROC curve.

Let *Y*_*i*_ = (*Y*_*i*, 1_, …, *Y*_*i*, *k*_)^′^ denote the *k*-dimensional vector of multiple correlated tests; such that *Y*_*i*, *k*_ denote the diagnostic result of the *k*th test (*k* = 1,…, *K*) when applied to subject *i* in a random sample of 523 subjects generated from normal distributions. Adjusting the covariates, the eq. 1 can be generalized on the latent true disease status as follows:

2 $$ {\displaystyle \begin{array}{cc}{d}_i\sim \mathrm{Bernoulli}\left({\pi}_i\right)& \left(i=1,2,\dots, 523\right)\\ {}\left(\left.{Y}_i\right|{d}_i,{x}_i\right)\sim \mathrm{MVN}\left(\mu \left({x}_i,{d}_i\right),{\Sigma}_{d_i}\right)& \end{array}} $$

where probability of being diseased (*π*) follows a logistic model:

$$ p\left(d=1\right)=\pi =\frac{\mathit{\exp}\left({\alpha}_s{\mathrm{x}}_{is}\right)}{1+\mathit{\exp}\left({\alpha}_s{\mathrm{x}}_{is}\right)}={\alpha}_0+{\alpha}_1{\mathrm{x}}_{i1}+\dots +{\alpha}_s{\mathrm{x}}_{is} $$

where *α* = (*α*_0_, *α*_1_, …, *α*_*s*_)^′^ is the vector of coefficients. Because we found that maternal age and BMI may play an important role in helping to discern GDM status, these variables utilized as disease and test covariates. x_*i*_ = (1, x_*i*1_, …, x_*is*_)^′^ indicates the covariate vector of an individual. The test scores follow a multivariate normal (MVN) distribution. The model for disease status and the three marker results for GDM data are given by:

$$ \mathrm{logit}\left(P\left({d}_i=1\right)\right)={\alpha}_0+{\alpha}_1\kern0.2em \mathrm{maternal}\kern0.2em {\mathrm{age}}_i+{\alpha}_2\kern0.2em {\mathrm{BMI}}_i $$

$$ E\left({Y}_k\right)=\mu \left(\mathrm{x},d\right)={\beta}_0^k+{\beta}_1^k\kern0.2em \mathrm{maternal}\kern0.2em {\mathrm{age}}_i+{\beta}_2^k\kern0.2em {\mathrm{BMI}}_i+{\beta}_3^k\kern0.2em {\mathrm{disease}}_i+{\beta}_4^k\kern0.2em \left({\mathrm{disease}}_i\times \mathrm{maternal}\kern0.2em {\mathrm{age}}_i\right)+{\beta}_5^k\kern0.2em \left({\mathrm{disease}}_i\times {\mathrm{BMI}}_i\right), $$

where ${\beta}^k=\left({\beta}_0^k,{\beta}_1^k,{\beta}_2^k,{\beta}_3^k,{\beta}_4^k,{\beta}_5^k\right)$ is the corresponding vector of regression coefficients.

For generating the composite test, a linear combination of the biomarkers (*Y*_*i*_^∗^ = *a*^′^*Y*) was employed. The optimal vector of linear combination is calculated as *a* = (Σ_0_ + Σ_1_)^−1^Δ(x) in which Δ(x) = *μ*(x, 1) − μ(x, 0). The combined AUC (cAUC) based on combined test scores can be estimated as $\Phi \left(\sqrt{a^{\prime}\Delta \left(\mathrm{x}\right)}\right)$. In addition, the covariate-adjusted combined ROC (cROC) curve for a given cut-off point value c is constructed by computing

$$ \left(1-c\mathrm{Spesificity}\left(\mathrm{c}|\mathrm{x}\right),c\mathrm{Sensitivity}\left(c|\mathrm{x}\right)\right)=\left(1-\Phi \left(\frac{c-{a}^{\prime}\mu \left(\mathrm{x},0\right)}{\sqrt{a^{\prime }{\Sigma}_0a}}\right),\Phi \left(\frac{a^{\prime}\mu \left(\mathrm{x},1\right)-c}{\sqrt{a^{\prime }{\Sigma}_1a}}\right)\right). $$

We independently specified $\mathrm{MVN}\left(0,\mathrm{I}{\sigma}_{\alpha}^2\right)$ prior for *α* in which I is the identity matrix, $\mathrm{MVN}\left(0,\mathrm{I}{\sigma}_k^2\right)$ prior for *β*^*k*^, and Wishart(*ν*, Γ) prior for Σ_*d*_ such that *ν* and Γ are degrees of freedom and scale matrix, respectively. To examine the convergence of the MCMC samples, autocorrelation plots and Geweke’s diagnostic test were used. Further, optimal marker combination for making diagnosis identified based on the largest estimated AUC.

For the Bayesian data analysis, the software package R2OpenBUGS in R software was made (https://cran.r-project.org/web/packages/R2OpenBUGS). Likewise, for the Geweke diagnostic, we used the coda library in R (http://www-fis.iarc.fr/coda). After obtaining the parameter estimates, differences in maternal age and BMI variables between GDM groups were evaluated using a Mann-Whitney U test. *P* values less than 0.05 were considered statistically significant. The statistical programing R software, version 3.5.1, was utilized for the univariate analyses (http://www.rproject.org).

## Results

In total, the data from 523 pregnant women with mean (SD) age of 28.76 (±5.33) years were analyzed. The range of maternal age at childbirth was between 25 and 40 years. The mean (SD) BMI was 24.57(±3.22) kg/m^2^. Additionally, the mean (SD) uE3, β-hCG, and AFP was 1.06 (±0.58) MOM, 1.17 (±0.77) MOM, and 1.11 (±0.43) MOM, respectively. Likewise, all the biomarkers’ values followed the normal distribution (*p* > 0.05).

The results of fitting Bayesian LCM for estimating the diagnostic accuracy parameters for each biomarker are provided in Table 1. According to Table 1, the posterior means of Sensitivity, Specificity, and AUC for uE3 were 67% (95% CrI [0.58–0.72]), 86% (95% CrI [0.81–0.88]), and 0.65 (95% CrI [0.56–0.69]), respectively. Moreover, the estimated Sensitivity, Specificity, and AUC were 78% (95% CrI [0.70–0.84]), 82% (95% CrI [0.79–0.85]), and 0.62 (95% CrI [0.54–0.68]), respectively, for β-hCG. Finally, for AFP, Sensitivity, Specificity, and AUC were estimated as 71% (95% CrI [0.66–0.78]), 92% (95% CrI [0.89–0.98]), and 0.58 (95% CrI [0.51–0.62]), respectively. Additionally, the estimated Δ for uE3 (0.29), β-hCG (0.37), and AFP (0.32) showed that there was reasonable separation between distribution of diseased and non-diseased groups.

**Table 1:** Sensitivity, Specificity, and AUC of each biomarker under Bayesian latent class model assuming without a gold standard

Biomarker	Parameters	Mean	Median	SD	MC error	95% CrI^a^
uE3						
	Sensitivity	0.67	0.66	0.15	0.02	0.58–0.72
	Specificity	0.86	0.88	0.002	0.0007	0.81–0.88
	AUC	0.65	0.65	0.12	0.01	0.56–0.69
β-hCG						
	Sensitivity	0.78	0.77	0.20	0.03	0.70–0.84
	Specificity	0.82	0.82	0.003	0.0007	0.79–0.85
	AUC	0.62	0.62	0.18	0.04	0.54–0.68
AFP						
	Sensitivity	0.71	0.70	0.06	0.002	0.66–0.78
	Specificity	0.92	0.93	0.06	0.002	0.89–0.98
	AUC	0.58	0.58	0.07	0.002	0.51–0.62

*uE3* unconjugated estriol, *β-hCG* beta-human chorionic gonadotropin, *AFP* alfa-Fetoprotein, *SD* standard deviation, *MC* monte carlo, *CrI* credible interval, *AUC* area under receiver operating characteristic curve

**Fig. 1:**
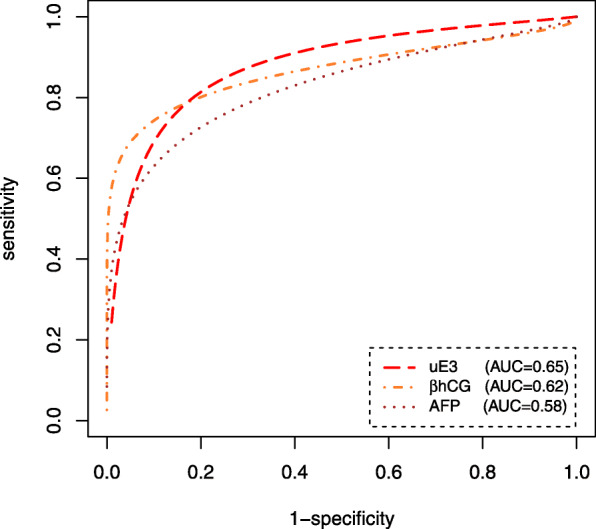
Estimated Bayesian ROC curve for each biomarker in the absence of a gold standard

Also the estimated ROC curve and the corresponding area under the ROC curve based on the Bayesian LCM for each biomarker are presented in Fig. 1.

**Fig. 2:**
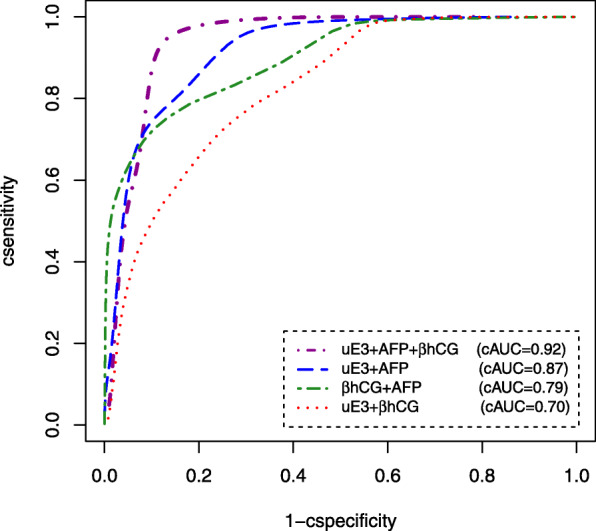
Estimated Bayesian cROC curve for combination of the three serum markers without a gold standard

The Bayesian estimates of the diagnostic accuracy indices for combination of biomarkers adjusting for maternal age and BMI are summarized in Table 2. Based on Table 2, in combination of uE3 and β-hCG results, the cSensitivity, cSpecificity, and cAUC were estimated as 68% (95% CrI [0.64–0.75]), 66% (95% CrI [0.61–0.77]), and 0.70 (95% CrI [0.62–0.78]), respectively. Moreover, the estimated cSensitivity, cSpecificity, and cAUC for combination of uE3 and AFP results were 76% (95% CrI [0.70–0.82]), 72% (95% CrI [0.66–0.78]), and 0.87 (95% CrI [0.81–0.91]), respectively. Furthermore, for combination of AFP and β-hCG results, the cSensitivity, cSpecificity, and cAUC were estimated as 72% (95% CrI [0.68–0.81]), 75% (95% CrI [0.68–0.84]), and 0.79 (95% CrI [0.71–0.87]), respectively. Ultimately, cSensitivity, cSpecificity, and cAUC for combining all the three biomarkers were 94% (95% CrI [0.91–0.99]), 86% (95% CrI [0.80–0.92]), and 0.92 (95% CrI [0.87–0.98]), respectively. Meanwhile, plots of Bayesian cROC curves for various combinations of biomarkers are given in Fig. 2.

**Table 2:** The estimated diagnostic accuracy parameters for composite test from Bayesian latent class model adjusting for maternal age and BMI

Biomarker	Parameter	Mean	Median	SD	MC error	95% CrI
uE3 and β-hCG						
	cSensitivity	0.68	0.66	0.14	0.01	0.64–0.75
	cSpecificity	0.66	0.65	0.22	0.02	0.61–0.77
	cAUC	0.70	0.70	0.11	0.01	0.62–0.78
uE3 and AFP						
	cSensitivity	0.76	0.78	0.36	0.01	0.70–0.82
	cSpecificity	0.72	0.71	0.15	0.01	0.66–0.78
	cAUC	0.87	0.87	0.14	0.02	0.81–0.91
AFP and β-hCG						
	cSensitivity	0.72	0.74	0.42	0.05	0.68–0.81
	cSpecificity	0.75	0.75	0.23	0.01	0.68–0.84
	cAUC	0.79	0.78	0.21	0.02	0.71–0.87
uE3 and β-hCG and AFP						
	cSensitivity	0.94	0.95	0.06	0.001	0.91–0.99
	cSpecificity	0.86	0.86	0.15	0.02	0.80–0.92
	cAUC	0.92	0.91	0.11	0.01	0.87–0.98

*uE3* unconjugated estriol, *β-hCG* beta-human chorionic gonadotropin, *AFP* alfa-fetoprotein, *SD* standard deviation, *MC* monte carlo, *CrI* credible interval, *cSensitivity* combined Sensitivity, *cSpecificity* combined Specificity, *cAUC* combined area under receiver operating characteristic curve

Apparently, comparing the diagnostic accuracy indices tells us that combination of all the three biomarkers has resulted in remarkable improvement in predicting GDM (Fig. 2). Based on this optimal composite diagnostic test, 483 (92.4%) of all study participants were assigned to the GDM group. Also, without GDM group consists of 40 (7.6%) of 523 study participants. Of note, the mean (SD) maternal age of pregnant women with GDM was significantly higher than those without GDM (31.95 ± 4.34 years vs. 29.53 ± 4.18 years, *p* = 0.021). Furthermore, the mean (SD) BMI was significantly higher for pregnant women with GDM compared with those without GDM (23.92 ± 2.37 kg/m^2^ vs. 22.38 ± 2.24 kg/m^2^, respectively, *p* < 0.001).

## Discussion

Gestational diabetes mellitus is one of the most common medical problems during pregnancy. According to previous publications, it is associated with increased risk of perinatal morbidity and mortality. Thus, using convenient modality is of great importance for screening and early diagnosis of this disease. As noted earlier, the main disadvantage of the gold standard for detection of GDM is the fact that it should be measured almost at the end of the second trimester gestation [32]. This delay in diagnosis might lead to increased risk of developing various diseases. However, this study was an attempt to assess the ability of combination of three biomarker results by calculating the Bayesian estimation of the sensitivity, specificity, and AUC in the early second trimester of pregnancy in the absence of GS.

The findings from the current study showed that none of the biomarkers alone could predict GDM. More clearly, because of the low values of AUCs (0.65, 0.62 and 0.58), the ability of uE3, β-hCG and AFP for discriminating women with and without GDM was not sufficient. Thus, we investigated whether linear combination of the three test results could lead to improved diagnostic performance by maximizing the area under the ROC curves comparing with a single marker. Among all different combinations of biomarker results, the findings of the used Bayesian LCMs exhibited that the combination of the three markers had the highest accuracy for detecting GDM while adjusting for maternal age and BMI. More clearly by this combination, 94% of pregnant women with GDM could be correctly forecasted. In this regard, so far different perspectives have examined the relationship between GDM and the biomarkers. For instance, Raty et al. evaluated maternal serum β-hCG and AFP levels between 117 pregnant women with GDM at 14 to 18 weeks of gestation. They showed statistically significant difference in these biomarker levels between the control and GDM groups [33]. Additionally, another study in Turkey has indicated that β-hCG was a weak predictor of GDM in weeks 11 to 13 of gestation [34]. Also, Gurram et al. found a significant relationship between β-hCG levels in GDM groups between 11 and 13 weeks of gestation in women who underwent first trimester aneuploidy screening [17]. In contrast, in a cross sectional study by Spandana et al., they found no significant difference in β-hCG between two groups of GDM from 11 to 13 weeks of pregnancy [15]. Also Sancken and Bartels in 2001 demonstrated that there was no significant difference in AFP level between healthy subjects and those with GDM during 15 to 20 gestational weeks [35]. However, Thornburg et al. reported a significant relationship between AFP and GDM at 14–20 weeks’ gestation [36]. A recent study by Hur et al. established the relationship between β-hCG, uE3, AFP and GDM in the early second trimester of pregnancy. They showed that, after controling for age and maternal weight, uE3 and β-hCG were useful predictors of GDM development [16].

In the context of evaluating the diagnostic accuracy of the biomarkers, we found only two published papers. In a study conducted by Sayn et al., the sensitivity, specificity, and AUC for AFP were computed as 32.3, 78%, and 0.51, respectively; 69.6, 47.9%, and 0.56 for hCG, respectively; and 36.2, 78.5%, and 0.57 for uE3, respectively in the second trimester of pregnancy [14]. Likewise, Kavak et al. has also reported a sensitivity, specificity and AUC of 57.5, 59%, and 0.58 for β-hCG, respectively, in the first trimester [18]. These two studies have used classical methods rather than advanced statistical techniques for determining the diagnostic accuracy parameters. Moreover, both evaluated the ability of the biomarkers in the presence of the GS test. Eventually, in these two recent studies, the diagnostic performance of the biomarkers was assessed individually. On the other hand, the values of test accuracy indices were relatively low. Unlike these studies, our findings suggest that combination of β-hCG, uE3, and AFP biomarkers, in addition to adjusting the covariates, could result in good predictor for early detection of GDM in the absence of a GS. Evidently, using additional information including covariate information may be helpful in mitigating the lack of a GS and better discriminatory accuracy. Notably, we recommend that the clinicians investigate the pathophysiologic mechanism between β-HCG, uE3 and AFP in future research.

The model presented in the current study was latent class model. There is a large body of evidence on this topic over the past decades. This model does not work well when there is considerable overlap between distributions of test results. To be more specific, if the overlap between the distributions of test values for the diseased and non-diseased populations become too large, assigning the correct disease status in the overlapping region will be difficult [28]. Based on our findings, the overlap between the distributions of two groups was not large for all three biomarkers (Δ = 0.29, 0.37, and 0.32). Hence, it seems that the presented model is appropriate for the analysis of the available data.

In this paper, we employed a Bayesian method with non-informative priors for the assessment of composite test in detecting GDM independent of a GS. For all parameters, since the MC errors were small and also the CrIs were narrow, we can conclude that the estimates were accurate. One of the benefits of Bayesian method is that there is no need to know the actual disease status of the participants. Meanwhile, the approach is not limited to unnatural choice of prior distributions. In fact, it can be a valuable generalization of the frequentist methods which allows for incorporation of prior information about test accuracy in the population under the study [37, 38]. It is worth noting that the Bayesian method, unlike the restrictions of the frequentist intervals, can provide credible intervals with acceptable coverage properties [39]. In the current study, the MCMC algorithm was applied to draw a random sample from the joint posterior distribution. There are some reasons for using this algorithm which as follows. In the Bayesian approach, obtaining the posterior estimator of each parameter by means of a numerical integration method is very difficult. Additionally, complexity of the joint posterior distribution and high dimensional integral problem made the direct calculation impossible. To overcome the mentioned problems, the Gibbs sampling algorithm based on MCMC methods was employed [40]. Extensive literature is available on diagnostic accuracy analysis for scenario involving absence of perfect reference standard information [31, 41–44]. For example, Collins and Huynh reviewed frequantist and Bayesian approaches for assessing the ability of various types of diagnostic tests (i.e. binary, ordinal, and continuous) without a perfect reference standard [42]. In agreement with our findings, all of these researches believe that the inference within the Bayesian framework can provide more reliable estimates of diagnostic test accuracy.

To the best of our knowledge, this is the first study that proposes a general Bayesian LCM based on MCMC algorithms for evaluating the performance of combining uE3, β-hCG, and AFP for early detection of GDM. An advantage of the methodology is that it allows the evaluation of accuracy of a screening test or combination of multiple screening tests without a perfect reference standard. Nevertheless, there are several limitations in our study that should be considered. First, the presented methods are based on the normal assumption for the test values. Often, for many diagnostic tests, an appropriate transformation is required to confirm the normality assumption. For the situations in the absence of knowledge about the true disease status of the individuals, the transformation is less straightforward and cannot guarantee the normality. Secondly, we had some missing information in patients’ records such as the patients’ disease history, hemoglobin A1C (HbA1C), Blood pressure (BP) and family history in a self-report way. Thirdly, due to the cross-sectional nature of the study design, causal inferences could not be made. To overcome the first problem, one can use nonparametric approach or skewed distributions. This is an interesting topic that could be examined in our future work.

## Conclusions

An oral glucose tolerance test as a GS is recommended for screening of GDM between the 24th and 28th gestational weeks. Nevertheless, the screening should be performed earlier in pregnancy for high-risk women. In summary, the findings of the current literature disclosed that the diagnostic accuracy of combination of the three serum markers’ values is desirable for predicting GDM when no information about the GS test is available in the early second trimester of pregnancy. The early detection along with adequate treatment and also evaluation of intervention strategies might reduce some diabetes-related complications in pregnancy outcome for mother and her child.

## Data Availability

The datasets analysed during the current study are not publicly available due to the reasonable risk that study participants may be identified. The datasets presented in this study may be available from the corresponding authors on reasonable request.

## Abbreviations

GDM: Gestational diabetes mellitus
GS: Gold standard
OGTT: Oral glucose tolerance test
LCM: Latent class model
β-hCG: Beta-human chorionic gonadotropin
uE3: Unconjugated estriol
AFP: Alfa-fetoprotein
AUC: Area under receiver operating characteristic curve
CrI: Credible interval
ROC: Receiver operator characteristic
MOM: Multiples of median
BMI: Body mass index
SD: Standard deviations
SPSS: Statistical Package for the Social Sciences
MCMC: Markov chain monte carlo
MC: Monte Carlo
MVN: Multivariate normal
cROC: Combined ROC
cAUC: Combined AUC
cSen: Combined Sensitivity
cSp: Combined Specificity
HbA1C: Hemoglobin A1C
BP: Blood pressure
